# Associations between patient portal use and electronic health record (EHR) data timeliness in type 2 diabetes mellitus care

**DOI:** 10.1007/s40200-024-01468-6

**Published:** 2024-07-31

**Authors:** Kevin Wiley, Justin Blackburn, Eneida Mendonca, Nir Menachemi, Mary De Groot, Joshua R. Vest

**Affiliations:** 1 Department of Healthcare Leadership and Management, Medical University of South Carolina (MUSC), 151-B Rutledge Avenue, Charleston, SC USA; 2 Department of Health Policy and Management, Richard M. Fairbanks School of Public Health, Indiana University, Indianapolis, IN USA; 3 University of Cincinnati, Cincinnati, OH USA; 4 Diabetes Translational Research Center, School of Medicine, Indiana University, Bloomington, USA

**Keywords:** Telemedicine, Chronic disease, Data quality

## Abstract

**Objective:**

Patient data is subject to missingness and errors. Patient portals enable patients managing type 2 diabetes mellitus (T2DM) to review and correct data to avoid retesting, medication errors, and diagnostic mistakes. We examined whether patient portal use was associated with electronic health record (EHR) data timeliness in T2DM care.

**Research Design and methods:**

We analyzed EHR data from a panel of adult patients to determine whether portal use improved data timeliness. EHR data timeliness is measured as the number of days between patient encounters, accounting for mean attribute update periods, where available EHR attribute updates for T2DM measurements were present, including body mass, weight, glycated hemoglobin A1c, cholesterol, blood pressure, serum creatinine, and smoking status. We performed negative binomial regressions with fixed effects to estimate the association between patient portal use and EHR data timeliness. Sensitivity analyses were conducted using Poisson regressions.

**Results:**

Nearly a third (31.3%) of patients in our sample actively used the health portal. There were fewer days (111.9 days vs. 136.7 days; *p* < 0.001) between EHR attribute updates for patients who used health portals compared to patients who did not. Data timeliness was lower among female, non-Hispanic White Medicare beneficiaries. Based on regression analyses, portal use was associated with an expected 3.6 (*p* < 0.001) percentage point decrease in days between attribute updates, indicating improved EHR timeliness.

**Conclusion:**

Improving the quality of health information may streamline decision-making in partnership with patients who produce data points across clinical settings. Active use of patient portals and digital health tools in chronic disease care are critical for care management and clinical decision-making, especially for patients managing type 2 diabetes across clinical settings.

**Supplementary Information:**

The online version contains supplementary material available at 10.1007/s40200-024-01468-6.

## Introduction

Type 2 Diabetes (T2DM) care relies on a wide array of electronic health record (EHR) data elements collected across providers and settings, including laboratory results, prescription information, and behavioral indicators (e.g., smoking status) [1]. These numerous touchpoints and differential documentation practices may affect the EHR data quality [2]. Errors and missing patient information can have costly care coordination ramifications if uncorrected [3]. Adverse outcomes from missing patient information, particularly in the primary care setting, include duplicate medications, missed or delayed diagnoses, missed immunizations, and repeat testing and procedures [4, 5].

Patient portals enable bidirectional communication between providers and patients to share and receive health information for critical care processes [6]. Approximately 44 million patients have access to ambulatory care health information documented and maintained in their patient portal [7]. With this technology, patient portals serve as a means to improve data quality by allowing patients to view, verify, and correct their records where information may be missing, erroneous, or repeated [7, 8]. The opportunity to improve EHR data quality via patient portals may optimize care coordination and self-management, which are critical to improving chronic disease care outcomes [6, 9, 10]. Likewise, there may be potential outcome improvements among older and underrepresented patient populations due to historically low digital literacy, low patient portal use, and lower levels of EHR data quality [11–13].

Although patient portals enable data verification and correction, some barriers may impede tangible improvements in overall data quality. Namely, prior research has identified challenges limiting patients’ access and consumption of their health information as a result of disparate documentation patterns, health system capacity, and inaccessible patient portals [14, 15]. Data documentation standards may be broadly available, but time drafting care summaries or for other notation in the EHR may vary across providers [15]. Despite these challenges, health portals have allowed patients to correct mistakes in diagnoses, medical history, medications, and test results [10–13]. Patient portal use has also been found to increase overall patient engagement by enabling access to their health information to manage various aspects of their health [9, 16]. For example, health portals empower patients with relevant information and wherewithal that reduce care fragmentation for patients [17]. While prior research has investigated the implications of digital health tool use on data quality dimensions in clinical settings [18]., studies have not examined associations between patient portal use and EHR data timeliness in T2DM care. Thus, rigorously examining the effect of portal use on the quality of patient data in a chronic disease domain is important to understanding variations in timely access to relevant, actionable information.

### Objective

Our study examined the relationship between patient portal use and EHR data timeliness for patients managing T2DM. We quantified a measure of timeliness using structured EHR data. Although patient portal use is becoming more common, access barriers still limit portal activation and use among some disadvantaged patient groups who may benefit most [11]. Therefore, we specifically examined associations between patient portal use and EHR data quality stratified by patient race, age, sex, and insurance status.

## Methods

### Study design

We examined the association between patient portal usage and EHR data timeliness in a panel of patients aged ≥ 18 years between 2017 and 2021.

### Sample & setting

Our study sample was derived from two urban large health systems in central Indiana. These systems operate more than 30 outpatient and specialty facilities. Specialists in these health systems provide approximately a third of outpatient care. Patients were included in the sample if they were diagnosed with T2DM by a primary or specialty care provider between their index and final encounter. Encounters in this study were derived from outpatient settings, where patients are commonly seen for routine chronic disease care.

### Data

We extracted patient demographic, encounter, and laboratory data from the Indiana Network for Primary Care (INPC), a statewide health information exchange (HIE) data repository, for patients seen at any facility affiliated with two large health systems in central Indiana between January 2017- July 2021. The INPC was established in 1994 as a repository for 38 health systems, 19,095 practices, and 19 million patients [19]. This HIE maintains health information for five major health systems, county and state public health departments, and Medicaid data in the state of Indiana.

### Outcome variable: EHR data timeliness

Timeliness was defined as the age of data elements representing a patient’s health at a desired time of interest [20, 21]. We quantified EHR data timeliness as the number of days between patient encounters where available EHR attribute updates for T2DM measurements were present, including body mass index, body weight, glycated hemoglobin A1c, cholesterol, blood pressure, serum creatinine, and smoking status. Fewer days between EHR attribute updates indicate better timeliness at the time of the patient encounter [22].

### History of Patient Portal Use

We created a dichotomous measure of active patient portal use determined as patients who received and opened a secure message sent by a health care provider or provider organization. Patients without a history of active patient portal use did not receive or open a secure message sent by a healthcare provider or organization. Under this definition, patients who had been issued a patient portal account but had never used the technology were considered non-users.

### Demographic variables

We included patient characteristics identified in EHR data for subgroup analyses. The patient characteristics included age, race, sex, and insurance status. Age categories were measured as (1) 18–25; (2) 26–45; (3) 46–55; (4) 56–65; and (5) > 65. Race was categorized as (1) non-Hispanic White, (2) African American/Black, (3) Hispanic; and (4) Other. We modeled patients’ sex as (1) Female and (2) Male. Lastly, insurance status is categorized as (1) Commercial, (2) Medicare, (3) Medicaid, (4) Self-Pay, and (5) Other.

### Analytic methods

We computed descriptive statistics and cross-tabulations using frequencies, percentages, means, and standard deviations. One-way ANOVA analyses and chi-square tests examined bivariate relationships between independent and dependent variables. Negative binomial regressions with fixed effects estimated the association between patient portal use and EHR data timeliness. We used negative binomial models as appropriate for the over-dispersed, count-based nature of the outcome variable (i.e., the total number of days). Analyses accounted for linear time trends for 2017–2021 using yearly time dummy variables. Hausman tests were performed to determine the proper model fit [23]. We fit separate regressions to examine variations in patient portal use and EHR data timeliness by patient age, race, sex, and insurance status. For all models, we reported marginal effects estimates. Statistical analyses were performed using Stata 16.0 (Stata Corp., College Station, TX, USA). The institutional review board (IRB) at Indiana University reviewed and approved this research protocol.

### Sensitivity analyses

To check the robustness of our findings, we undertook several sensitivity analyses. First, we repeated analyses by fitting fixed effects Poisson models with time dummies. Poisson regressions were fitted to test whether the magnitude of the effects were different given similar data distributions for the outcome variable. Second, to check that the results were not the product of extreme values, we fit separate fixed-effects regression where we truncated the dependent variable, EHR data timeliness, at the 90th and 95th percentiles. We tested these approaches to determine if there were variations in higher values of EHR data timeliness. We tested a different measure of timeliness using a quotient that accounts for the mean attribute update times between patient encounters [21]. Prior research utilized a timeliness quotient to capture changes in specific attribute updates due to the expiration or obsolescence of some tests. Lastly, we adjusted our main analysis to account for pre- and post-COVID-19 status using a binary indicator. These indicators were assessed to determine whether workflow disruptions affected documentation and data capture processes in T2DM care.

## Results

### Descriptive analyses

The study sample included 35,759 patient-encounter date observations. The average age was 53.6 years. Most patients in the study were documented as non-Hispanic Black (47.4%), female (65.8%), and had a Charlson comorbidity score of 1 or higher (50.8%) (Table 1).

**Table 1:** Descriptive patient characteristics and EHR Data Timeliness

	EHR Data Timeliness for All Patients			
	(%)	Mean Timeliness (in days) (SE)	95% CI	P value
Patient portal use				< 0.001
Yes	31.3	111.9 (19.0)	73.9-149.8	
No	68.7	136.7 (27.6)	81.8-191.6	
Patient Sex				< 0.001
Female	65.8	120.9 (23.8)	73.5-168.3	
Male	34.2	128 (23.7)	80.9-175.1	
Patient Race/Ethnicity				0.004
White	28.3	112.9 (25.1)	63.1-162.8	
African American/Black	47.4	129.5 (22.3)	85.0-173.9	
Hispanic	16.6	158.5 (56.4)	46.2-270.8	
Other	3.9	42.8 (16.6)	9.6–75.9	
Age				0.007
18–25	5.7	105.3 (36.5)	32.5-178.1	
26–45	39.3	155.4 (33.6)	88.4-222.3	
46–55	25.8	107.9 (27.9)	52.2-163.5	
56–65	20.1	101 (26.9)	47.5-154.5	
> 65	6.7	113 (52.6)	8.5-217.9	
Charlson Comorbidity Score				0.021
0	26.9	165 (47.2)	71.3-259.2	
1	50.8	115.5 (19.4)	86.9-154.1	
2	17.3	121.8 (42.0)	38.2-205.5	
3	4.1	71.2 (22.3)	26.9-115.4	
Insurance				< 0.001
Commercial	25.2	136.6 (42.6)	51.7-221.5	
Medicare	24.1	81.9 (16.5)	49.1-114.8	
Medicaid	41.4	134.4 (23.9)	86.6-182.1	
Self-pay	6.0	151.6 (48.7)	54.6-248.6	
Other	3.2	15.5 (5.5)	4.5–26.5	
COVID-19 Status				< 0.001
Pre-COVID-19	67.2	66.5 (0.3)	65.9–67.1	
Post-COVID-19	32.8	71.2 (0.5)	70.2–72.1	
Year				< 0.001
2017	42.3	112.2 (15.4)	81.5-142.9	
2018	21.9	152.4 (45.9)	60.9-243.8	
2019	21.4	128.9 (49.9)	29.3-228.4	
2020	13.6	123.8 (41.6)	41.1-206.6	
2021	0.7	43 (8.3)	15.2–68.3	

**Fig. 1:**
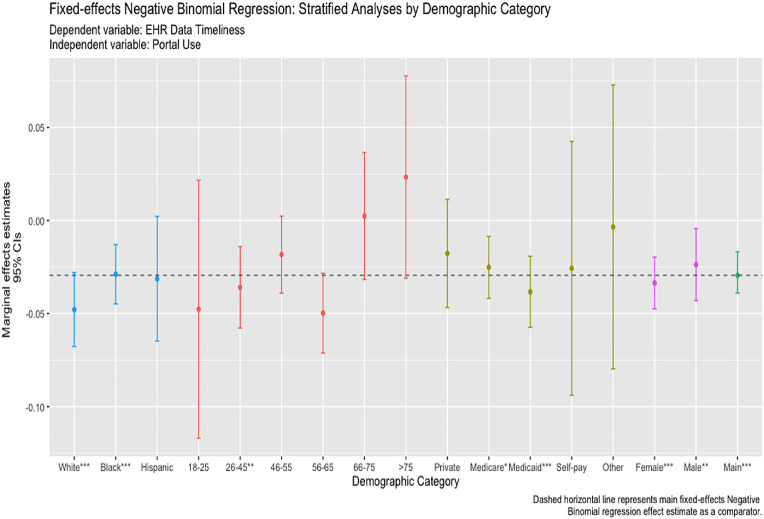
Mean Timeliness by Year and Patient Portal Use (2017–2021)

Nearly a third (31.3%) of the sample used the patient portal (Table 1). Mean EHR data timeliness, i.e., days between EHR attribute updates, was lower among patients who actively used patient portals (111.9 days) compared to patients who did not use patient portals at all (136.7 days; *p* < 0.001) (Table 1). Timeliness was lower among patients identified as female, non-Hispanic White, > 65 years of age, had a Charlson score of 3, and were insured by Medicare. EHR data timeliness improved among patient portal users during the study period (2017–2021) (Fig. 1).

### Association between patient portal use and data timeliness

**Fig. 2:**
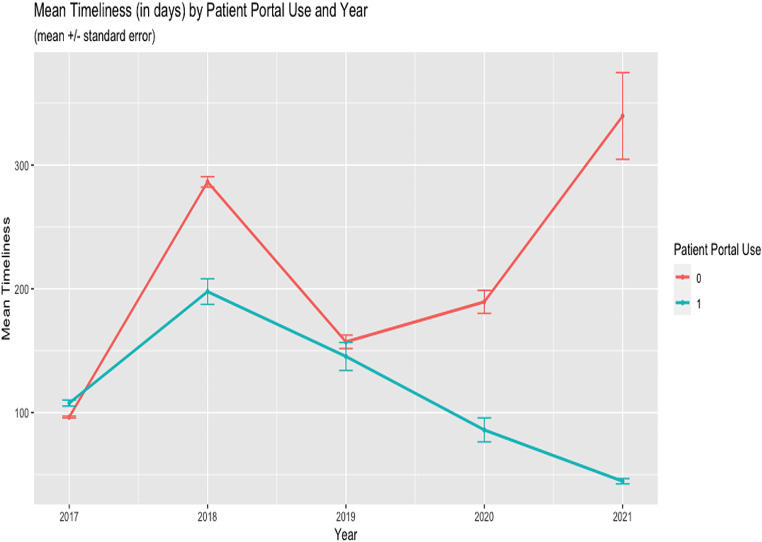
Stratified Fixed-effects Negative Binomial Regression Analyses: Race, Age, Insurance Status, Sex, and the Main Regression Model

Patient portal use was associated with an expected decrease in the EHR data timeliness (in days) of -0.036 (*p* < 0.001) (Table 2). That is, patient portal usage was associated with fewer days between relevant attribute updates in the EHR. In stratified analyses, patient portal usage was generally associated with timelier data for some patient groups. Specifically, patient portal use was associated with timelier data among female (ME= -0.041; *p* < 0.001) and male (ME= -0.029; *p* = 0.017) patients and among patients who were enrolled in Medicare (ME= -0.033; *p* = 0.003) and Medicaid (ME= -0.044; *p* < 0.001) (Fig. 2). Some of these effects indicated timelier data compared to the main effect. Patient portal use was associated with timelier data among non-Hispanic White patients (ME= -0.061; *p* < 0.001) and non-Hispanic Black patients (ME= -0.035; *p* < 0.001). Lastly, patient portal use was associated with timely EHR data among patients aged 26–45 (ME= -0.040; *p* = 0.001) and 56–65 (ME=-0.063; *p* < 0.001) (Fig. 2).

**Table 2:** Association between Patient Portal Use and EHR Data quality– conditional negative Binomial regression with fixed-effects (reported as marginal effects estimates)

	EHR Data Timeliness Model 1. Main Negative Binomial Regression with Fixed effects				
	ME	se	95% CI		P value
Patient Portal Use	-0.036	0.007	-0.049	-0.022	< 0.001
Year (ref: 2017)					
2018	0.088	0.009	0.071	0.105	< 0.001
2019	0.038	0.008	0.021	0.054	< 0.001
2020	0.029	0.009	0.012	0.047	0.001
2021	0.027	0.014	-0.007	0.055	0.056

### Sensitivity analyses

Sensitivity analyses were largely supportive of our primary analysis results. In Poisson and negative binomial regressions that examined outliers in the dependent variable at the 90th and 95th percentiles, patient portal use was associated with expected statistically significant decreases in the EHR data timeliness measures (i.e., more timely data) and was comparable to the primary model (Tables 3 and 4 in Appendix A). We modeled the dependent variable using a quotient to account for the mean time between updates to EHR data attributes, including body weight, body mass index (BMI), blood pressure, cholesterol, serum creatinine, glycated HbA1c, and smoking status (Table 5 in Appendix A). The quotient computes a timeliness value between 0 and 1. These results showed that patient portal use decreased days between attribute updates, albeit with smaller effect sizes due to the construction of the measure. Patients who used portals were also predicted to have fewer days between EHR attribute updates following the Public Health Emergency Declaration for the COVID-19 pandemic (Table 6 in Appendix A).

## Discussion

This study found that patient portal use was associated with timelier EHR data or shorter times between relevant EHR attribute updates. Further, findings from this study underscore the critical role health information access plays in improving overall data access and quality, which is critical for patient engagement and managing T2DM [9, 10, 24]. A considerable number of health information technology (HIT) policies have been developed to ensure that patients have access to their patient information to improve engagement and chronic disease management [25]. Prior research has examined how patient portal use improves engagement and self-management, and this work adds insights to the potential association with data quality [9, 16, 26].

Patient portal usage represents additional contact between the patient and the healthcare system for data collection and validation with healthcare providers [26–29]. Prior research has suggested that patient portal access and use enables data correction and verification [7]. Patient perceptiveness could explain the association between portal usage and timeliness observed in this study. Improving the timeliness and actionability of clinical data are goals for healthcare systems and current policy mandates for information sharing, as timely data are important diagnostic indicators in chronic disease management [25]. Additionally, the association between patient portal usage and timelier EHR data was consistent across numerous patient groups. Differential patient portal use outcomes among patient demographics raised concerns that such technological interventions may not benefit all patients [7, 27, 28] For example, research suggests lower usage of patient portals among the elderly and the uninsured [13, 29]. While our results align with this research, EHR data timeliness for relevant type 2 diabetes clinical indicators was moderately timelier among elderly patients.

In general, the benefits of using the patient portal were not fully realized among elderly patients. Generally, lower health and computer literacy levels are concerning among older age groups [13]. Also, chronic conditions are prevalent among these age groups, and older patients tend to utilize care more often than their younger counterparts. Thus, their care and data produced from these interactions are closely monitored. Healthcare organizations might consider increasing patient portal education among elderly populations and others who may not have experience or confidence in using digital health tools. This would significantly improve awareness of these tools and access to critical information that could support care among older patients. For example, effective interventions should consider psychosocial and socio-technical barriers to accessing patient portals, given these tools are often used outside of clinical settings to access information [13].

Healthcare organizations have increasingly adopted and used EHR technologies that support patient portal functionality to improve patient education and engagement [26–29]. The Meaningful Use (MU) program, sponsored by the federal government, incentivized the use of EHRs and patient portals to improve patient information access [30, 31]. These efforts were refined in the Centers for Medicare and Medicaid Services (CMS) through its Promoting Interoperability program [32]. The 21st Century Cures Act also established guidelines for improving patient portal access to optimize chronic disease management and reduce unnecessary healthcare interactions [32]. The effects of greater patient portal access and use on EHR data quality remain underexamined. Our findings provide evidence that patient portal use reduces time intervals between updates to relevant data. However, these results may be unique to patients closely monitored in chronic disease management programs and shifts from in-person care and test reporting to virtual delivery formats to limit disease exposure [33]. We observed effects during, before, and after the COVID-19 pandemic, indicating that for any change in patient portal use, the time between EHR attribute updates decreased slightly in the months following the Public Health Emergency Declaration.

Past research examined the relationship between a single measure of EHR data timeliness and care quality [34]. We sought to operationalize EHR data timeliness using multiple metrics and approaches to demonstrate measure applicability in future research that examines the quality of EHR data and its fitness and subsequent use. However, our methodology is not exhaustive nor conclusive to chronic disease domains. Further research is needed to examine other factors contributing to variations in attribute update times and how adopting and using health information technologies like patient portals and EHRs mitigate them. For example, active use of patient portals may also improve EHR data completeness in chronic disease settings [35]. Still, improvements may vary in non-chronic disease settings and across provider types where the informed presence of disease indicators is less discernable.

### Limitations

There are several limitations in this study. First, we measured timeliness in terms of days between updates. The biomedical informatics literature has identified case-based definitions for timeliness and other data quality measurements [20, 36]. While research has examined other dimensions of EHR data quality (i.e., completeness, concordance, plausibility) [34, 37]., we focused on a single dimension of EHR data quality. Second, we may be unable to generalize to other conditions and data types. We specifically selected T2DM patients because patients managing chronic disease have more frequent visits and clinical measurements. Patient groups relying on fewer diagnostic measures or those of shorter duration may not see the same associations in timeliness from patient portal use. Future research should account for multiple timeliness measures. Additionally, other data quality dimensions should be explored to fully understand how patient portals improve the veracity and comprehensiveness of patient data. Researchers should also test these methods in other clinical domains, like cancer care.

## Conclusion

Active patient portal use may reduce EHR data timeliness in chronic disease care processes. Improving timeliness may streamline decision-making in partnership with patient populations who produce data points across clinical settings. Active use of patient portals and other digital health technologies may bolster improvements in overall data quality.

## Electronic Supplementary Material

Below is the link to the electronic supplementary material.

Appendix A - Sensitivity Analyses

## Data Availability

Patient electronic health record (EHR) data were used in this study and are not publicly available.
