# Supplementary material for: Associations between patient portal use and electronic health record (EHR) data timeliness in type 2 diabetes mellitus care
Source: J Diabetes Metab Disord. 2024 Jul 31;23(2):2073–80. doi: 10.1007/s40200-024-01468-6 (PMC11599696; doi:10.1007/s40200-024-01468-6)
Supplement: Supplementary file 1 — Appendix A - Sensitivity Analyses [file 40200_2024_1468_MOESM1_ESM.docx]

**Appendix A. Sensitivity Analyses**

Table 3. Sensitivity Analyses for Examining the Relationship between Patient Portal Use and EHR Data Timeliness - Conditional Negative Binomial Regression with Fixed-effects Truncated at the 90th Percentile

|  | EHR Data Timeliness Truncated at the 90th Percentile  Model 1. Negative Binomial Regression with Fixed effects | | | |
| --- | --- | --- | --- | --- |
|  | ME | 95% CI | | P value |
| Patient Portal Use | -0.025 | -0.036 | -0.015 | <0.001 |
| Year (ref: 2017) |  |  |  |  |
| 2018 | 0.439 | 0.031 | 0.057 | <0.001 |
| 2019 | 0.015 | 0.003 | 0.028 | 0.017 |
| 2020 | 0.014 | 0.001 | 0.027 | 0.043 |
| 2021 | 0.034 | 0.015 | 0.055 | 0.002 |
| Constant | 0.561 | 0.549 | 0.549 | 0.574 |

Table 4. Sensitivity Analyses for Examining the Relationship between Patient Portal Use and EHR Data Timeliness - Conditional Negative Binomial Regression with Fixed-effects Truncated at the 95th Percentile

|  | EHR Data Timeliness Truncated at the 95th Percentile  Model 1. Negative Binomial Regression with Fixed effects | | | |
| --- | --- | --- | --- | --- |
|  | ME | 95% CI | | P value |
| Patient Portal Use | -0.059 | -0.069 | -0.049 | <0.001 |
| Year (ref: 2017) |  |  |  |  |
| 2018 | 0.054 | 0.041 | 0.067 | <0.001 |
| 2019 | 0.012 | -0.002 | 0.025 | 0.077 |
| 2020 | -0.004 | -0.018 | 0.009 | 0.542 |
| 2021 | -0.019 | -0.042 | 0.004 | 0.101 |
| Constant | 0.245 | 0.232 | 0.257 | <0.001 |

Table 5. Sensitivity Analyses for Examining the Relationship between Patient Portal Use and EHR Data Timeliness - Conditional Negative Binomial Regression with Fixed-effects using Timeliness Quotient (See Appendix 9)

|  | EHR Data Timeliness Quotient Accounting for Mean Attribute Update Time  Model 1. Fixed-effects Regression model | | | |
| --- | --- | --- | --- | --- |
|  | me | 95% CI | | P value |
| Patient Portal Use | -0.000 | -0.000 | -0.000 | 0.800 |
| Year (ref: 2017) |  |  |  |  |
| 2018 | -0.000 | -0.000 | -0.000 | <0.001 |
| 2019 | -0.000 | -0.000 | -0.000 | <0.001 |
| 2020 | 0.000 | -0.000 | -0.000 | <0.001 |
| 2021 | 0.000 | -0.000 | -0.000 | <0.001 |
| Constant | 0.001 | 0.001 | 0.001 | 0.001 |

Table 6. Patient Portal Use Pre- and Post-COVID-19 Emergency Declaration
Dependent Variable: EHR Data Timeliness

|  | Fixed-effects Negative Binomial  Regression for EHR Data Timeliness | | | |
| --- | --- | --- | --- | --- |
|  | ME | 95% CI | | P value |
| COVID-19 Status (ref: Pre-COVID-19) |  |  |  |  |
| Post-COVID-19 | -0.016 | -0.028 | -0.004 | 0.011 |
